# Traditional Chinese medicine, Fuzheng Kang-Ai decoction, inhibits metastasis of lung cancer cells through the STAT3/MMP9 pathway

**DOI:** 10.3892/mmr.2021.12244

**Published:** 2021-06-25

**Authors:** Longmei Li, Sumei Wang, Xiaobin Yang, Shunqin Long, Shujing Xiao, Wanyin Wu, Swei Sunny Hann

Mol Med Rep 16: 2641-2648, 2017; DOI: 10.3892/mmr.2017.6905

Following the publication of the above article, an interested reader drew to the authors' attention that various of the data panels shown for the cell migration assay experiments in Figs. 2B and 3 appeared to show overlapping regions, such that they were not generated from discretely performed experiments. The authors have re-examined their original data, and realize that the figures in question were assembled incorrectly. In addition, the authors have also realized that certain of the western blotting data panels in Figs. 5 and 6 had likewise been assembled incorrectly.

The corrected versions of Figs. 2, 3, 5 and 6 are shown on the next two pages. All these corrections were approved by all authors. The authors regret that these errors were included in the paper, and are grateful to the Editor of *Molecular Medicine Reports* for allowing them the opportunity to publish this corrigendum. They also wish to emphasize that the errors made during the compilation of the figures did not substantially alter any of the major conclusions reported in the study, and apologize to the readership for any inconvenience caused.

## Figures and Tables

**Figure 2.**
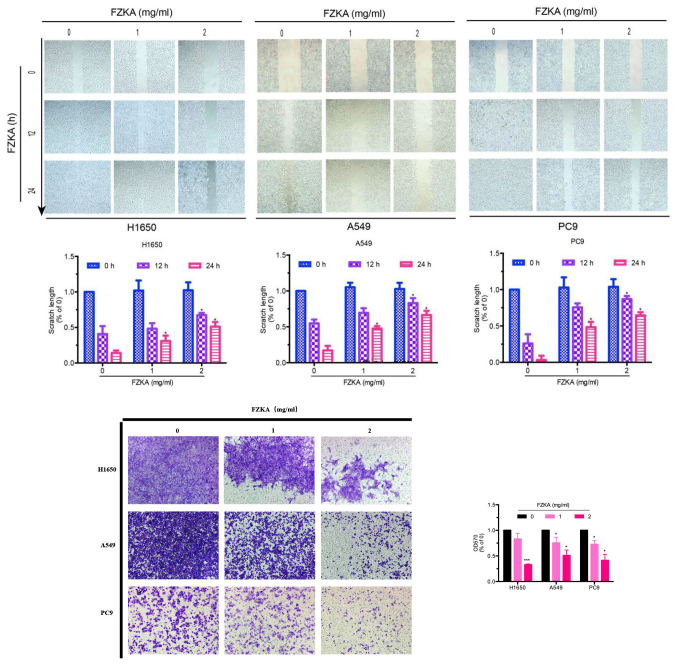
FZKA inhibits lung cancer cell migration. (A) H1650, A549 and PC9 cells were cultured (4×10^5^) in 6-well plates, and were incubated until cell density reached 90%. Cell monolayers were wounded by scratching with a 200-µl pipette tip and were treated with FZKA (0, 1 and 2 mg/ml). Results are representative of three independent experiments. (B) H1650, A549 and PC9 cells were plated in a Transwell plate. The lower chamber was filled with 500 µl cell culture medium containing 30% fetal bovine serum. Cells were diluted to 0.5×10^6^/ml and were pretreated with FZKA (0, 1 and 2 mg/ml), after which a 200 µl cell suspension was added to the upper chamber and was incubated for 16 h. Magnification, ×40. *P<0.05 and ***P<0.001 vs. control (0 FZKA mg/ml). FZKA, Fuzheng Kang-Ai; OD, optical density.

**Figure 3.**
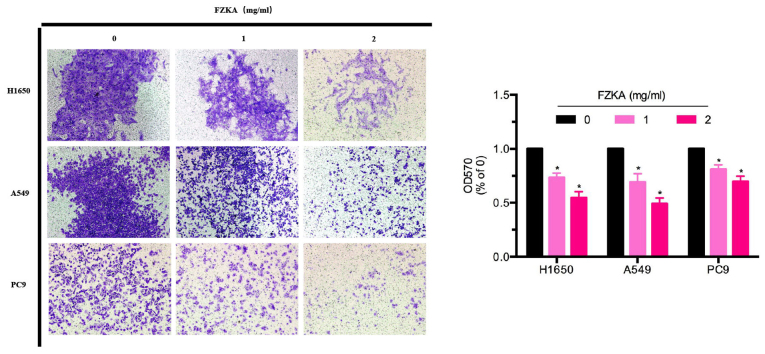
FZKA inhibits lung cancer cell invasion. H1650, A549 and PC9 cells were plated in a Transwell plate. Matrigel was injected into the upper chamber and 500 µl cell culture medium with 30% fetal bovine serum was added to the lower chamber. Cells were diluted to 0.5×10^6^/ml and were pretreated with FZKA (0, 1 and 2 mg/ml), after which a 200 µl cell suspension was added to the upper chamber and was incubated for 16 h. Absorbance was measured at 570 nm using a microplate reader. The experiment was repeated three times. Magnification, ×40. *P<0.05 vs. control (0 FZKA mg/ml). FZKA, Fuzheng Kang-Ai; OD, optical density.

**Figure 5.**
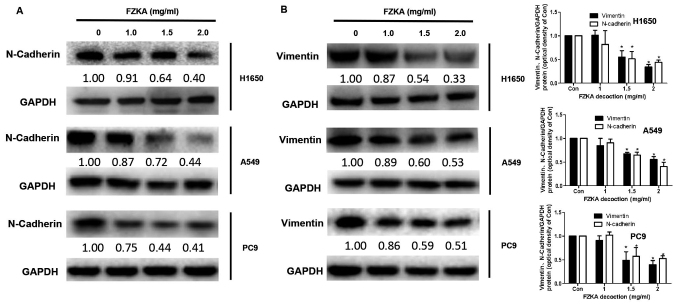
FZKA inhibits epithelial-mesenchymal transition in lung cancer cells. Protein expression levels of (A) N-cadherin and (B) vimentin were detected in lung cancer cells following treatment with FZKA by western blotting. Data were measured by ImageJ software. The experiments were repeated three times. *P<0.05 vs. control (0 FZKA mg/ml). FZKA, Fuzheng Kang-Ai.

**Figure 6.**
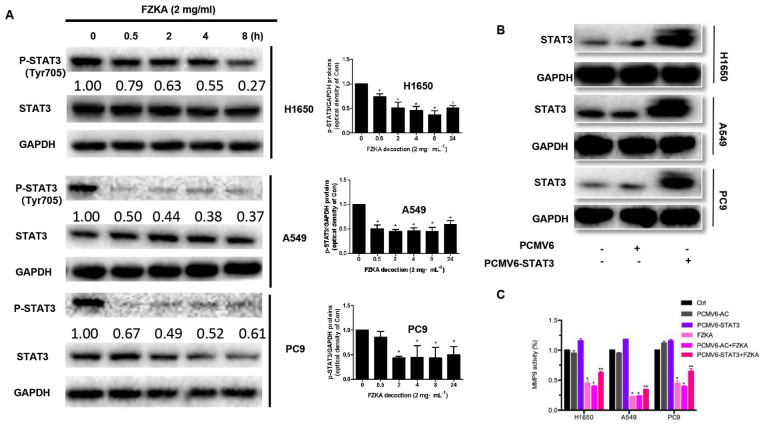
STAT3 regulates MMP9 activity in lung cancer cells treated with FZKA. (A) Protein expression levels of STAT3 were reduced following treatment with FZKA (2 mg/ml; 0, 0.5, 2, 4 and 8 h). *P<0.05 vs. 0 h. (B) To overexpress STAT3, cells (H1650, A549 and PC9) were seeded into 6-well plates, and transfected with pCMV6-AC (negative control) and pCMV6-AC-STAT3 DNA constructs, prior to treatment with FZKA. STAT3 protein expression was then measured by western blot analysis. (C) MMP9 activity was increased by STAT3 overexpression. Following treatment with FZKA, the FZKA-mediated inhibition of MMP9 activity was significantly suppressed by STAT3 overexpression. *P<0.05 and **P<0.01 vs. control (Ctrl). FZKA, Fuzheng Kang-Ai; MMP9, matrix metalloproteinase 9; STAT5, signal transducer and activator of signaling 3; Ctrl, control.

